# Predicting Film Genres with Implicit Ideals

**DOI:** 10.3389/fpsyg.2012.00565

**Published:** 2013-01-07

**Authors:** Andrew McGregor Olney

**Affiliations:** ^1^Institute for Intelligent Systems, University of MemphisMemphis, TN, USA

**Keywords:** film, genre, topic model, Netflix, likability, ideal, category, concept

## Abstract

We present a new approach to defining film genre based on implicit ideals. When viewers rate the likability of a film, they indirectly express their ideal of what a film should be. Across six studies we investigate the category structure that emerges from likability ratings and the category structure that emerges from the features of film. We further compare these data-driven category structures with human annotated film genres. We conclude that film genres are structured more around ideals than around features of film. This finding lends experimental support to the notion that film genres are set of shifting, fuzzy, and highly contextualized psychological categories.

## Introduction

Film genre theory has some outstanding problems both in terms of definition and analysis. Stam (2000) identifies two problems of definition in genre theory as circularity and the monolithic assumption. The problem of circularity arises when one tries to define a genre in terms of features like those given in Table 1.

**Table 1:** **Genre features adapted from Chandler (1997)**.

Feature	Example
Time	Films of the 1930s
Author	Stephen King
Age of audience	Kid film
Technology	Animated
Star	Sylvester Stallone
Director	Quentin Tarantino
Structure	Narrative
Ideology	Christian
Culture of origin	Bollywood
Subject matter	Disaster film
Location	Western

A feature-based analysis requires first assembling all the films representative of that genre and then analyzing their features. The problem of course is that this assumes the genres of the films are already known, where in fact the analysis is being conducted to determine the genres in the first place. Thus the analysis is based on circular reasoning. The second problem of definition is the monolithic assumption, in which a film is assumed to belong to one and only one genre. While the monolithic assumption in some ways makes the task of genre definition simpler, it nevertheless ignores genres that are part of our public discourse, e.g., *romantic comedy*, which are intuitively hybrid genres.

The above discussion of film genre has parallels to the so-called classical view of categories in the cognitive psychology literature if film genre is considered analogous to a category. The classical view (see Smith and Medin, 1981; Murphy, 2004 for reviews) is perhaps most strongly associated with the idea that categories can be defined in terms of necessary and sufficient conditions, i.e., the minimal conditions which all members of the category must meet in order to belong. In practice, it is difficult to isolate the necessary and sufficient conditions required by the classical view. Consider the category *bachelor*. This is often defined by the necessary and sufficient conditions of *male* and *unmarried*. However true this may be, is it the case that a newborn baby is a bachelor? Or a teenager? Age/sexual maturity intuitively seems to be a so-called non-defining feature of the category *bachelor*. However there is evidence that humans use non-defining features in categorization tasks. In an experiment asking participants to judge whether two animals were in the same or different categories (Caramazza et al., 1976) found that the reaction times were correlated with previously obtained ratings of semantic distance. When the ratings of semantic distance were decomposed into two dimensions using multi-dimensional scaling, the two dimensions *ferocity* and *size* were significantly correlated with reaction time, *r*(28) = 0.45, *p* < 0.05. Thus the implication is that these two non-defining dimensions are used by participants when making same/different category judgments, a finding that is not easily accommodated by the classical view.

Additionally, the classical view predicts that category members belong to a category to the same degree. As a result, the semantic distance between the category *bird* and category items like *robin*, *goose*, and *ostrich*, should be the same. In other lines of research, this category to item relationship is referred to as typicality because participants are asked to rate how typical an item is of its category (Rosch and Mervis, 1975). Semantic distance has been shown to be highly correlated with typicality, *r*(46) = 0.89, *p* < 0.01 (Rips et al., 1973), though some researchers prefer to consider them as separate constructs (Caramazza et al., 1976). Contrary to the prediction of the classical view, typicality ratings tend to reveal a graded structure to a category (Smith et al., 1974; Rosch, 1975), such that *robin* is considered a more typical *bird* than *goose*. Just as semantic distance and reaction time has been correlated (Caramazza et al., 1976), typicality has been correlated with reaction time, priming, and speed of learning (Rosch, 1975, 1978). In a series of experiments that parallel the decomposition of semantic distance into underlying dimensions (Rosch and Mervis, 1975) found that typicality effects can be predicted by the distribution of underlying features in that category. Specifically, the most typical members of a category have the most features in common with other members in the category and the least features in common with members of contrasting categories. For example, common features of *bird* include flying, dull coloring, and being about the size of a shoebox. A *robin* has all these properties, but less typical birds like *chicken*, *cockatoo*, or *ostrich* do not. Such family-resemblance effects have been found not only in natural categories but also artificial categories consisting of letter strings, indicating that these effects are not restricted to natural categories. Collectively, these findings suggest that film genre should have a graded structure consistent with typicality and non-defining dimensions. If so, this would be undermine the view of film genres as classical categories with necessary and sufficient conditions.

In a significant extension of Rosch and Mervis’s (1975) work (Barsalou, 1985) considered so-called ideals as determinants of typicality structure. An ideal is a salient dimension defining the success of a goal. For example, the category *birthday present* has the associated ideal *make the receiver happy*. Unlike natural taxonomic categories like *bird*, a *birthday present* can have arbitrary physical features and is largely only defined by its socio-cultural context. Since the notion of ideal as a determinant of typicality was introduced, a variety of effects have been found. Barsalou (1985) collected typicality ratings and correlated these with ratings of ideals for a variety of categories. These ideals differed for each category, but 7 out of 18 were either liking, enjoying, or fun. In particular, the category *things to do for weekend entertainment* was more strongly correlated with the ideal *enjoyment* than other measures like family-resemblance and frequency. Ideals influenced typicality more for goal-directed categories like *birthday present* than taxonomic categories like *bird*. A second experiment showed that ideals could causally affect typicality ratings by manipulating participants’ ideals for artificial categories. Additional studies have shown that expertise seems to increase the influence of ideals on typicality in taxonomic categories like *fish* (Burnett et al., 2005) and *trees* (Lynch et al., 2000). So ideals not only structure goal-directed categories like *entertainment*, but they also can structure taxonomic categories like *fish* when expertise is high.

Given these findings from the cognitive psychology literature on categories, it may be futile to apply the classical view of categories to film genre. To do so would assume that film genres are somehow unlike the wide variety of categories previously studied. It therefore appears reasonable to expect semantic distance and typicality effects with respect to film genre, whether the drivers for such effects are based on family-resemblance or are based on ideals. However, implementing a family-resemblance categorization experiment for film genre falls prey to the same problems of genre theory mentioned above: circularity and the monolithic assumption. Whereas cognitive psychology experiments may take for granted the natural category like *bird* and ask participants how typical a member like *robin* is of that category, such clear demarcations are not available in the domain of film. In film, the categories themselves are not clear cut and without controversy. Since a feature-based analysis would require presuppositions about categories in order to collect typicality ratings, other analyses are warranted.

Rather than utilize an experimental procedure that presupposes the existence of genre-type categories, as a feature-based analysis would, we identify two possible experimental procedures that can be used to examine the status of genre-type categories without presupposing their existence. The first of these has been used previously (Voorspoels et al., 2011) and follows (Caramazza et al., 1976): assemble a large number of film-film pairs, gather similarity ratings, and project the ratings into a low-dimensional structure using multi-dimensional scaling. The second approach, which we follow in this paper, instead mirrors the notion of ideal: rather than compare the film to another film, ask the participants to compare the film to their ideal of a film. It should be noted that this operationalization of ideal is distinct from Barsalou (1985) where participants were asked to rate according to the ideal specified by the experimenter. Since the participants’ ideals are not specified, we call them *implicit ideals*.

We take this alternative approach to genre by analyzing *likability ratings* (Olney, 2010). Likability ratings are commonplace: many web sites, e.g., Amazon, allow users to rate item likability or their overall satisfaction, without explicitly asking them to compare the item to a category or giving them a ideal-based criterion to rate against. Thus likability ratings reflect implicit ideals, where the criterion used varies across participants and items being rated. While the ratings datasets of many websites are kept confidential, some companies like Netflix have released large datasets containing hundreds of thousands of ratings by hundreds of thousands of users (Netflix, 2010). In Netflix ratings, as with Amazon ratings, users are allowed to rate whatever films they choose on a 1–5 star scale, according to whatever criteria they wish.

In this paper, we analyze the ideals implicit in likability ratings to build a model of film genre consistent with a psychological category. Our studies focus on the ratings from the Netflix dataset, which we incorporate into a probabilistic topic model (Griffiths et al., 2007). Our new approach defines genre based on likability ratings of content rather than the features of content. By incorporating likability ratings into a topic model, we generate intuitively plausible categories that are predictive of human annotated genres (Studies 1–2; Olney, 2010). We compare the likability model with a likability model using unary vectors, two different feature-based models, and a likability model with binary weightings (Studies 3–6). In all cases, the likability model exhibits superior prediction of human annotated genres. We conclude that film genres are structured more around ideals than around features of film. This finding lends experimental support to the notion that film genres are set of shifting, fuzzy, and highly contextualized psychological categories.

## Study 1: Likability-Based Topic Model

Before attempting to predict human annotated genres with likability-based topics, it was necessary to validate that likability-based topics will emerge under a topic model from the Netflix dataset. Previous support for this hypothesis can be found in Rubin and Steyvers (2009), where an extension of a topic model is used to predict user’s Netflix ratings. However, we wish to achieve the same result using a plain topic model without (Rubin and Steyvers, 2009)’s additional order-logit model to generate a user’s rating, i.e., we wish to show that a topic model can be used to derive genres by itself.

Topic models (Griffiths and Steyvers, 2002; Griffiths et al., 2007), also known in other communities as Latent Dirichlet Allocation (Blei et al., 2003), are a class of generative statistical models typically applied to text. Topic models ignore the order of words in a document, making them somewhat similar to methods such as latent semantic analysis (LSA; Landauer et al., 1998, 2007), however there are significant differences. Rather than reduce the dimensionality of the data according to an optimal least-squares approximation, topic models use a probabilistic model that assumes the data was generated by an underlying process involving hidden variables. While LSA expresses the data along latent dimensions, i.e., singular vectors, which have no clear semantic interpretation, topic models express the data according to the topics that generated the data, and these topics are expressed as a collection of semantically related words, i.e., the words that are most probable given a topic. Thus these topics possess a graded membership structure that parallels typicality effects in the psychological categorization literature.

For example, consider a newspaper article about farming. The article implicitly contains topics like *plants*, *weather*, or *farm equipment*. These topics may not be equally represented, so the article might be 30% *plant*, 50% *weather*, and 20% *farm equipment*. Each of these topics has associated words in descending probability, e.g., *plants* may be 40% soybeans, 30% wheat, 20% weeds, and 10% grass. The article is made up of these individual words. What a topic model attempts to do is uncover the hidden topics in an article by comparing that article’s words to the words in many other articles. By looking at the patterns of words over many documents, the topic model can build a probabilistic model of what words belong together. Words that belong together correspond to topics.

More formally, the standard topic model makes the following assumptions. For each document, there is an associated distribution of topics. Each of these topics has an associated distribution of words. To generate a document, one first probabilistically samples from the distribution of topics, yielding a particular topic. One then probabilistically samples from the distribution of words associated with that particular topic, yielding a word. This process can be repeated to generate more words and more documents. In this way a topic model specifies how to *generate* the observed data. Conversely, a model may be *fitted* to existing data using probabilistic inference. Model fitting is accomplished by randomly initializing the model and then using Gibbs sampling to re-estimate the model’s parameters, iteratively, until the model converges. For more details (see Griffiths et al., 2008; Blei, 2012).

Though topic models have primarily been applied to text in the cognitive science community, the model itself is agnostic to the underlying data it represents, so long as that data has a form consistent with the assumptions of the model. One generalization of these assumptions would be as follows: data consists of a set of samples, each sample has a distribution of topics, and each item in the sample is generated from one of these topics. Using this intuition, it is fairly straightforward to map the Netflix dataset into a form consistent with the topic model, as we will further clarify below.

### Method

#### Data

The data used in this study consisted of the Netflix dataset, which is freely available online (Netflix, 2010). The dataset has a collection of information applicable to both training a model as well as evaluating the model using the Netflix API. In this study and succeeding studies, only the training data was used. The training data consists of two logical components. The first is a master file which lists for each film a unique ID, along with the title and release year for the film. The second component is a folder which contains, for each film ID, the set of ratings given to that ID by various users. Each rating is a triple consisting of user ID, rating, and date of rating. Each rating is an integral number from 1 to 5. There are 17,770 movies in the dataset, 480,189 users, and 100,480,507 ratings. The dataset is sparse, meaning that not every user has rated every film. These data were collected between October, 1998 and December, 2005, so the Netflix interface under which they were collected was different than the present interface. In this early period of Netflix, many ratings were for films the user had already seen outside of Netflix, in order for Netflix to propose new films. Since Netflix only introduced streaming in 2007, all ratings we either for films seen outside Netflix or for films shipped through the mail on DVD.

#### Procedure

We mapped the Netflix dataset to the topic model format in the following way. Each customer is a mixture of genres, and each genre is a distribution over movies. To transform the existing Netflix dataset using this mapping, we collect all of the movies seen by a customer. The number of stars given to each film is represented by an equal repetition of a film’s label. For example, if a customer only rated the film *Whale Rider* and gave it three stars, then the customer would be represented as (Whale Rider, Whale Rider, Whale Rider), analogous to a document containing the same word three times. Under the assumptions of this mapping and the underlying topic model, each star in a customer’s rating can be generated by a different genre. For example two stars of *Whale Rider* might be generated by the drama genre, and one star might be generated by the foreign film genre.

The inference algorithm to fit our model to the Netflix data is identical to that used in typical topic models. However, given the large size of the dataset and the widespread availability of multi-core processors, we have created and make publicly available our code for fast parallel topic models in the C# language^1^. Inference parameters were as follows. The number of topics was 50, the prior for topics appearing in a document (α) was 1, and the prior for words appearing in a topic (β) was 0.01. The α and β smoothing parameters are typical (Steyvers and Griffiths, 2007). The model was run for 200 iterations.

### Results and discussion

An initial inspection of the topics found by the model reveals intuitive genre-like categories, as displayed in Table 2. The intuitive appeal of these genres is consistent with word-based topics presented in the topic model literature. Each topic list is rank ordered by probabilistic membership. Therefore the first ranked film in each topic is the most probable film given that topic, and so on. This ranking is derived from the ϖ matrix of the topic model. Only the top five most probable films per topic are presented due to space limitations.

**Table 2:** **Fifty topics from likability data**.

Topic	Title	Topic	Title
1	Bowling for Columbine	2	The Mummy Returns
	Fahrenheit 9/11		Bad Boys II
	Whale Rider		Face/Off
	Super Size Me		Behind Enemy Lines
	Hotel Rwanda		Tomb Raider
3	Signs	4	Van Helsing
	Panic Room		I
	Insomnia		The Chronicles of Riddick
	Road to Perdition		The Bourne Supremacy
	The Ring		Spider-Man 2
5	Forrest Gump	6	Monty Python and the Holy Grail
	Saving Private Ryan		Monty Python’s Life of Brian
	Braveheart		Blazing Saddles
	The Shawshank Redemption		A Fish Called Wanda
	The Green Mile		Monty Python’s The Meaning of Life
7	The Two Towers	8	Apollo 13
	The Empire Strikes Back		Titanic
	The Return of the King		Men in Black
	The Fellowship of the Ring		Home Alone
	Return of the Jedi		The Matrix
9	Spirit: Stallion of the Cimarron	10	My Big Fat Greek Wedding
	Brother Bear		Sweet Home Alabama
	Treasure Planet		How to Lose a Guy in 10 days
	The Lion King 1 1/2		Pretty Woman
	Stuart Little 2		Legally Blonde
11	Ray	12	Buffy the Vampire Slayer: Ssn 1
	Finding Neverland		Buffy the Vampire Slayer: Ssn 3
	The Aviator		Buffy the Vampire Slayer: Ssn 2
	Spanglish		Buffy the Vampire Slayer: Ssn 4
	The Incredibles		Buffy the Vampire Slayer: Ssn 5
13	Barbershop	14	Spirited Away
	The Original Kings of Comedy		Princess Mononoke
	Love and Basketball		Hero
	Barbershop 2		Akira
	Antwone Fisher		Ghost in the Shell
15	The Royal Tenenbaums	16	Queer as Folk: Ssn 1
	Being John Malkovich		Queer as Folk: Ssn 2
	American Beauty		Queer as Folk: Ssn 3
	Pulp Fiction		Latter Days
	Memento		The Broken Hearts Club
17	Talk to Her	18	Pink Floyd
	Blue		Killing Me Softly
	Red		Led Zeppelin
	All About My Mother		Caligula
	Cinema Paradiso		The Beatles Anthology
19	Pieces of April	20	Terms of Endearment
	In America		On Golden Pond
	Before Sunset		Fried Green Tomatoes
	Girl with a Pearl Earring		Driving Miss Daisy
	House of Sand and Fog		Tootsie
21	The Sopranos: Ssn 1	22	The Twilight Zone: Vol. 2
	The Sopranos: Ssn 2		The Twilight Zone: Vol. 1
	Sex and the City: Ssn 2		Prime Suspect 1
	The Sopranos: Ssn 3		Horatio Hornblower
	Sex and the City: Ssn 1		The Twilight Zone: Vol. 16
23	GoodFellas	24	Mary Poppins
	Seven		Aladdin
	The Godfather		The Lion King
	The Usual Suspects		A Bug’s Life
	Reservoir Dogs		Willy Wonka and the Chocolate Factory
25	Primal Fear	26	Hitch
	The Game		Coach Carter
	Presumed Innocent		Sahara
	A Perfect Murder		The Longest Yard
	The Firm		Hostage
27	Catch Me If You Can	28	Girl
	Pirates of the Caribbean		Chasing Amy
	The Bourne Identity		Cruel Intentions
	The School of Rock		Romeo + Juliet
	Ocean’s Eleven		Dogma
29	The Player	30	The Fugitive
	The Grifters		Clear and Present Danger
	sex		Kiss the Girls
	The Crying Game		Along Came a Spider
	Glengarry Glen Ross		The Green Mile
31	13 Going on 30	32	Under Siege
	Cheaper by the Dozen		The Last Boy Scout
	Freaky Friday		Demolition Man
	Mean Girls		Eraser
	The Prince and Me		Commando
33	Friends: Ssn 1	34	Donnie Darko
	Friends: Ssn 4		The Office: Series 1
	Friends: Ssn 3		The Office: Series 2
	The Best of Friends: Vol. 1		Bottle Rocket
	Friends: Ssn 2		Requiem for a Dream
35	Weird Science	36	Rear Window
	Fast Times at Ridgemont High		Citizen Kane
	Sixteen Candles		Chinatown
	Planes		The Godfather
	Better Off Dead		To Kill a Mockingbird
37	Dirty Dancing	38	Family Guy: Vol. 1
	Pretty Woman		The Simpsons: Ssn 3
	Stepmom		Family Guy: Vol. 2
	Sister Act		The Simpsons: Ssn 4
	Patch Adams		The Simpsons: Ssn 2
39	Independence Day	40	Band of Brothers
	Con Air		Secondhand Lions
	Pearl Harbor		Blue Collar Comedy Tour
	Twister		Master and Commander
	The Patriot		Pirates of the Caribbean
41	The Outlaw Josey Wales	42	Man on Fire
	The Dirty Dozen		Taking Lives
	Pale Rider		Mystic River
	A Fistful of Dollars		Out of Time
	Patton		Cold Mountain
43	The Wrath of Khan	44	Hope Floats
	First Contact		Where the Heart Is
	The Fifth Element		While You Were Sleeping
	Aliens		Save the Last Dance
	The Abyss		My Girl
45	Arsenic and Old Lace	46	The Exorcist
	His Girl Friday		Halloween
	The Philadelphia Story		A Nightmare on Elm Street
	It Happened One Night		Carrie
	Singin’ in the Rain		The Exorcist: Restored Version
47	Die Hard	48	The Money Pit
	The Terminator		Ruthless People
	Lethal Weapon		Oh God!
	Ghostbusters		The War of the Roses
	Beverly Hills Cop		Doc Hollywood
49	Happy Gilmore	50	Sense and Sensibility
	American Pie		Elizabeth
	Tommy Boy		Shakespeare in Love
	Billy Madison		Chocolat
	Meet the Parents		Pride and Prejudice

Consistencies in Table 2 are evident. For example, Topic 1 could be considered documentaries or biographically inspired independent films, Topic 2 consists of action films that veer toward the fantastic, Topic 9 is made up of animated films directed at children, and Topic 10 lists romantic comedies. However, inconsistencies are also apparent. For example is *Bad Boys II* really as fantastic as a film about mummies? Or are Michael Moore films really that much like *Whale Rider*? Under this critical view, what can be gleaned from Table 2 is somewhat mixed. On the one hand, it is clear that some sense of genre can be driven by a likability-based topic model. The topics appear to have both family-resemblance and a graded structure of membership consistent with typicality effects. On the other hand, it is unclear to what extent these likability-based topics correspond to typical film genres. Without a correspondence-based evaluation, it is unclear whether the topics in Table 2 represent strong coherent categories or an observer bias toward any category that might make them coherent.

## Study 2: Predicting with Likability-Based Topics

To further understand the topics that emerged in Study 1, we conducted an additional study using these likability-based topics to predict human annotated film genres. If indeed the likability-based topics can predict film genres significantly more than chance, then the link between likability-based topics and actual film genres would be confirmed.

### Method

#### Data

To create a predictive model using likability-based genres, it was necessary to use a large dataset with human annotated genres for each film. The Internet film Database (IMDB) is a freely available database with human annotated genres. IMDB further contains an enormous amount of information for a given film, ranging from the director and year of release to less commonly known information such as the art department. Included amongst the hundreds of pieces of information associated with each film is a set of 28 genres, listed in Table 3.

**Table 3:** **IMDB genres**.

Documentary	Animation	Family	Sport
Crime	Drama	Mystery	Action
Sci-Fi	Comedy	Short	Game-show
Romance	Fantasy	Adventure	Music
Thriller	Biography	History	Musical
Horror	Adult	War	Film-noir
Reality-TV	Western	Talk-show	News

Each film in IMDB is associated with one or more of the genres in Table 3. For example, the biopic, *Ray* based on the story of musician Ray Charles, is labeled with *biography*, *drama*, and *music*. How these genre labels were generated for IMDB is not clear, and inter-rater reliability for these genres is not available.

#### Procedure

We followed a two step procedure. First, we aligned the film titles in the Netflix dataset with the IMDB genres. Unfortunately, this is less straightforward than it might first appear. The Netflix dataset is intentionally sparse, including only title, year, and ratings for each film. Thus we were required to align very sparse descriptions of a film (in Netflix) with very rich descriptions of a film (in IMDB) where items in the descriptions occasionally did not match as described below.

We used IMDbPy (2010), a Python-based software library, to query and search for film titles in IMDB. This search capability purposely returns more than single title in order to accommodate alternate title forms. Using IMDbPy, a correspondence requiring an exact match of both year and title yielded only 8,283 exact matches out of a possible 17,770. Relaxing the exact match requirement so that years match and titles match up to the colon yielded an additional 1,082 matches.

Inspection of the data reveals that failures to match have a variety of reasons. First, typographic conventions differ between datasets, such that a foreign film may have its original title spelling in one dataset and an Anglicized title in another, e.g., *Character* and *Character*. In addition, year information may be off by one between the two databases. Sequels and series are a particular problem, such that one database may precede the name of an episode with the name of the series, whereas the other does not. Some errors also exist in the matched films. It is possible, though rare, for two films to be released in the same year with the same name. For example, *Ray* the *documentary* of Ray Charles, appeared in the same year as a genre *short* of the same name. Finally, because to the inconsistencies with series naming conventions and the partial match strategy described above, some within-genre mismatches can occur, e.g., *Star Trek: Insurrection* and *Star Trek: First Contact*. However, the distribution of genres is very similar in both the matched set and the original set, as shown in Table 4. Additionally, the correlation between the proportional distributions for original and matched sets is very strong, *r* = 0.978.

**Table 4:** **Proportion of genres**.

Genre	Matched	Original	Genre	Matched	Original
Action	0.14	0.12	Horror	0.05	0.04
Adult	0.00	0.02	Music	0.02	0.02
Adventure	0.04	0.04	Musical	0.01	0.01
Animation	0.04	0.05	Mystery	0.01	0.01
Biography	0.03	0.02	News	0.00	0.00
Comedy	0.24	0.20	None (missing)	0.00	0.05
Crime	0.06	0.05	Reality-TV	0.00	0.00
Documentary	0.08	0.10	Romance	0.01	0.01
Drama	0.21	0.19	Sci-Fi	0.01	0.01
Family	0.02	0.02	Short	0.01	0.03
Fantasy	0.01	0.01	Sport	0.00	0.00
Film-noir	0.00	0.00	Talk-show	0.00	0.00
Game-show	0.00	0.00	Thriller	0.02	0.01
History	0.00	0.00	War	0.00	0.00
-	-	-	Western	0.01	0.01

Once the 9,249 films were paired, we used the WEKA toolkit (Hall et al., 2009) to build a set of predictive models. Each model uses as features only the distribution of topics associated with each film, i.e., a row vector from the topic model in Study 1. For example, position 1 would be the probability that a film belongs in genre 1, position 2 to probability a film belongs in genre 2, and so on for all 50 genres. For each model, the genre class to be predicted is the first genre listed by IMDB. This restriction is due to WEKA’s inability to perform multi-class classifications, and implies that overall performance of the models is significantly lower than would be the case if any genre label associated with a film was permitted as a correct answer.

This common data format was used by a set of the following five models. First, ZeroR, which predicts the most prevalent class, e.g., *comedy*. Whereas predicting each class equally would be the simplest non-intelligent baseline, ZeroR embodies the simplest intelligent baseline. In order to outperform ZeroR, a classifier must not only predict the most prevalent class well but also predict other classes well. Secondly, NaiveBayes, which assumes the features to predict a class are independent and uses Bayes Rule to construct a classifier. The *naive* assumption that each feature contributes independently to the classification of an item significantly reduces the complexity of the algorithm for massive data sets with large numbers of features. Thirdly, AdaBoostM1 uses an ensemble of weak learners, in this case a decision stump, using the boosting approach (Schapire, 2003). AdaBoost is a *boosting* approach because it uses a majority vote of an ensemble of weak learners. Given an initial equal distribution of weights to the training data, AdaBoost iteratively allocates increasing weight to incorrectly classified training examples. Thus AdaBoost *adapts* to the training data by giving increasing priority to the most difficult items. Fourthly, J48 is a decision tree whose internal branching on attribute values is constructed to maximally discriminate amongst the training data. At each step, J48 calculates the potential to discriminate, called *information gain*, for each feature in the training data. J48 creates a new node in the tree for the most discriminating feature, such that training examples are split based on a particular value of that feature, creating branches in the tree below that node. This process is applied recursively to grow the decision tree. And finally, IBk is an instance/exemplar based classifier, i.e., k nearest neighbors where k has been set to 10 neighbors. A new item is classified by finding its k nearest neighbors in the training data and assigning it the classification of the majority of those neighbors. These algorithms were selected because they represent a cross section of the most widespread and effective machine learning techniques (see Wu et al., 2007) for a review.

Each model was trained using 10-fold cross validation in which the dataset is divided into ten bins, and the model trained 10 times, using a different bin as test data each time. Significant differences were measured using a paired samples t-test, *p* < 0.5, corrected for the variability introduced by cross validation (Nadeau and Bengio, 2003).

### Results and discussion

The results of the likability-based predictive models are displayed in the second column of Table 7. Numbers shown indicate percent correct, aggregated across all genre categories. All significant differences are relative to the ZeroR model for each set.

Interestingly there is a fair distribution of performance across all models for predicted genres. The worst performer is NaiveBayes, worse than the ZeroR model, while the best performer is IBk-10, at 41%. All differences in this first set are significant.

Three important points are clear from this data. The first is that likability-based topics are indeed significantly better than chance at predicting human annotated genre categories. Indeed, the result is slightly stronger than this, since ZeroR predicts the most prevalent category, *comedy*, rather than a uniform probability of 1/28, or 4%. Thus it is more appropriate to say that likability-based topics are significantly better at prediction than a reasonably intelligent baseline predictor.

A second important result is that the likability-based topics are indeed strong and coherent, predicting the correct human annotated label in 41% of cases. This is almost double the ZeroR baseline of 23%. That a content-free analysis, based purely on likability ratings, can predict genres is surprising and provocative. Even more surprising is that likability-based topics can predict so well, especially given the stringent single-class criterion of correctness.

The third and final important point is the use of IBk-10 as a machine learning rule. IBk is perhaps the simplest machine learning rule. When presented with a new item to classify, IBk finds the k nearest neighbors of that item and then classifies the item according to the majority of neighboring items. IBk does not introduce any additional parameters or assumptions other than the parameter k, so the effectiveness of the IBk classifier can be wholly attributed to the topic model, which projected each film into a 50 dimensional subspace of genres.

However, given the perceived purity of genres in Table 2, it may be possible to accomplish the classification task with even fewer parameters. To explore this intuition, we reanalyzed the data by converting each 50 dimensional vector to a unary vector with a value of one at its maximal element and zero elsewhere. This unary representation removes the possibility of combinations of dimensions contributing to the performance of classification. Instead, only the strongest topic, the topic that is most associated with that film, has any influence on the genre classification.

The resulting IBk classifier is 38.63% accurate compared to the original 41.22% accuracy, indicating that virtually all of the information used to correctly classify each genre is given by the maximum corresponding topic, a one to one mapping. Of further interest are the kinds of errors made by the simpler classifier, many of which are intuitively reasonable. Table 5 presents a confusion matrix specifying the probability of a predicted genres for a given actual genre. Columns corresponding to genres that were never predicted have been omitted.

**Table 5:** **Probabilities of predicted genres in unary model**.

Correct genre	Probability of predicted genre						
	Documentary	Animation	Crime	Drama	Action	Comedy	Horror
Talk-show	1.00	0.00	0.00	0.00	0.00	0.00	0.00
News	1.00	0.00	0.00	0.00	0.00	0.00	0.00
History	0.73	0.00	0.00	0.00	0.00	0.27	0.00
Documentary	0.51	0.01	0.00	0.38	0.02	0.08	0.00
Family	0.04	0.75	0.00	0.06	0.01	0.14	0.01
Animation	0.01	0.62	0.00	0.04	0.22	0.11	0.00
Game-show	0.00	0.33	0.00	0.33	0.00	0.33	0.00
Music	0.07	0.01	0.00	0.89	0.00	0.04	0.00
Thriller	0.00	0.00	0.03	0.73	0.07	0.13	0.03
Sport	0.33	0.00	0.00	0.67	0.00	0.00	0.00
Drama	0.07	0.02	0.02	0.52	0.07	0.28	0.02
Short	0.13	0.06	0.01	0.54	0.09	0.16	0.01
Horror	0.00	0.01	0.00	0.52	0.06	0.04	0.36
Romance	0.02	0.00	0.00	0.49	0.14	0.34	0.01
Sci-Fi	0.00	0.08	0.00	0.49	0.17	0.19	0.06
Crime	0.04	0.00	0.09	0.46	0.06	0.33	0.02
Biography	0.16	0.00	0.01	0.45	0.03	0.34	0.01
Adult	0.03	0.00	0.06	0.39	0.16	0.32	0.03
Fantasy	0.03	0.04	0.00	0.27	0.21	0.23	0.22
War	0.07	0.00	0.00	0.14	0.71	0.07	0.00
Western	0.06	0.00	0.00	0.22	0.59	0.13	0.00
Action	0.02	0.04	0.02	0.33	0.41	0.16	0.02
Film-Noir	0.00	0.00	0.00	0.10	0.00	0.90	0.00
Reality-TV	0.32	0.00	0.00	0.05	0.00	0.58	0.05
Comedy	0.04	0.04	0.00	0.30	0.04	0.56	0.02
Musical	0.06	0.03	0.00	0.42	0.00	0.48	0.00
Mystery	0.02	0.00	0.10	0.35	0.10	0.44	0.00
Adventure	0.07	0.19	0.01	0.20	0.19	0.33	0.02

The results of the unary model suggest that the likability-based topics displayed in Table 2 may properly be thought of as likability-based genres. The one to one correspondence shows that genre identity is not distributed across a vector of 50 topic probabilities. Instead, genre is highly associated with a single, unweighted topic.

## Study 3: Predicting with Content-Based Features

A counter claim could be made that while likability ratings can reveal genres, they are not as effective at predicting human annotated genres as a more traditional, content-based analysis. This claim parallels the previously found distinction of categories based on family-resemblance as opposed to categories based on ideals (Barsalou, 1985). In Study 3, we compare content-based predictive models with the likability-based predictive models of Study 2.

### Method

#### Data

The same 9,249 films were evaluated as in Study 2. However, the content-based models use as features a collection of information from IMDB, chosen to best match the features sometimes used by film critics to determine the genre of a film, as described in Table 1. These features are listed in Table 6.

**Table 6:** **IMDB features**.

Feature	Type
Plot	Numeric
Title	Numeric
Actor 1	Nominal
Actor 2	Nominal
Director	Nominal
Year	Numeric
MPAA	Nominal

A few features of Table 6 warrant brief remarks. Plot is a plot synopsis of the film, generated by an IMDB user. Sometimes films have multiple synopses generated by different users; these are concatenated into one synopsis. The two actor features are the first and second named actors on the billing, i.e., the stars of the film. MPAA is the rating of the film, e.g., PG-13. The other features are self-explanatory.

#### Procedure

Some of these features are nominal, such as actor and director names, meaning that they are associated with a fixed set of labels as is genre in Table 3. However, the IMDB plot synopsis is an arbitrary string of considerable length, e.g., 500 words, and the title is a shorter but equally arbitrary string. In order to be usable features that two films could have in common, both plot and title were transformed using term frequency/inverse document frequency (tf*idf; Manning and Schütze, 1999). Tf*idf is used by search engines to decide how important words in a document are. Words that occur frequently in a document are weighted more (tf); however, words that occur in many documents are weighted less (idf). Using this procedure, each word in the string became its own feature. Because the number of words features was very large, two methods were used to prune the space. First, stop words were removed. These are common words that have low informational value like articles, pronouns, prepositions, and auxiliary verbs. Secondly, stemming was used on each word. Stemming is an approximate process of suffix removal designed to reduce a word to its base or root form, e.g., “laughed” to “laugh.” Using stop words and stemming reduced the original word features to 1,420 features. Thus each synopsis was converted into a numeric vector of 1,420 elements, each of which corresponds to the weighted occurrence of a stemmed word in the synopsis. Since not all words occur in every synopsis, many elements will be zero. The WEKA command line used to convert plot and title to these numeric features was “StringToWordVector -R1,2 -W100 -prune-rate-1.0 -C -T -I -N0 -L -S -SnowballStemmer -M1 -WordTokenizer.”

As in Study 2, the genre class to be predicted is the first genre listed by IMDB. In order to be directly comparable to Study 2, the same set of five models was used, i.e., ZeroR, NaiveBayes, AdaBoostM1, J48, and IBk, with the same set of parameters. Likewise each model was trained using 10-fold cross validation, and significant differences were measured using a corrected paired samples t-test, *p* = 0.05.

### Results and discussion

The results of the content-based predictive models are displayed in Table 7 along with the parallel likability-based results from Study 2. Numbers shown indicate percent correct, aggregated across all genre categories. All significant differences are relative to the ZeroR model for each set.

**Table 7:** **Results in percent correct**.

Model	Likability-based	Content-based	Synopsis-based
rules.ZeroR	23.51	23.51	23.51
bayes.NaiveBayes	9.94	27.12	14.36
meta.AdaBoostM1	23.96	23.51	23.51
trees.J48	37.30	29.21	25.20
lazy.IBk	41.22	27.50	29.89

Performance on the content-based models is worse than the performance on the likability-based set. For content-based models, there is very little deviation away from ZeroR. All differences are significantly different from ZeroR, except AdaBoostM1, which is not significantly different from ZeroR. The best model of the content-based set, J48, has only 29% accuracy compared to 41% for IBk in the first set. This performance is particularly poor considering the base rate (ZeroR) is 23%.

It should be stressed that user-generated plot synopses are quite similar to the methodology commonly used in generating family-resemblance data. Both tasks involve generating salient characteristics of items from memory. The plot synopses contain salient aspects of the film such as narrative structure and character development. In previous work Rosch and Mervis (1975) asked participants to list the attributes of a common object, e.g., a *bicycle* has the attribute *wheels*, and the resulting family-resemblance data was strongly correlated with category structure. If film genre is determined by family-resemblance, the plot synopsis data should be a powerful predictor for correct categorization.

However, our results indicate that likability-based topics are more predictive of human annotated genres than content-based features. The content-based models are only 67% as accurate as likability-based models. From this we conclude that likability-based topics appear to have greater power in explaining human genre categorization behavior than do more traditional feature-based models. This finding is consistent with the hypothesis that film genre is determined more by ideals than by family-resemblance.

## Study 4: Synopsis-Based Topic Model

An alternative explanation to the poor performance of content-based models relative to likability-based models in Study 3 is that the content-based models were subject to a “feature explosion.” Under this alternative hypothesis, the content-based models were harmed by a large number of non-predictive features, i.e., noise, while the likability-based models were helped by the dimensionality-reduction performed by the topic model. To further investigate this alternative explanation, we created a topic model of the synopses used in Study 3. Only the synopses were used because they are the richest single source of family-resemblance information used in the previous study, and synopses are well aligned with the typical methodology for generating family-resemblance data.

### Method

#### Data

The same synopses were used as in Study 3. The synopses came from the 9,249 films in the IMDB database that had been matched to films from the Netflix dataset in Study 2.

#### Procedure

Each synopsis was cleaned of punctuation and lowercased. A stop list was used to remove approximately 500 of the most common words in English. Additionally, user email addresses, used to identify the author of the synopsis, were removed to prevent biasing of the results. A standard topic model was created using each synopsis as its own document, with the following parameters identical to Study 1. The number of topics was 50, the prior for topics appearing in a document (α) was 1, and the prior for words appearing in a topic (β) was 0.01. The α and β smoothing parameters are typical (Steyvers and Griffiths, 2007). The model was run for 200 iterations. In this way the same parameters were used for the likability-based topic model and the synopsis-based topic model.

### Results and discussion

As in Study 1’s likability-based topic model, an inspection of the topics found by the synopsis-based topic model reveals intuitive categories, as displayed in Table 8. Each topic list is rank ordered by probabilistic membership, i.e., *evil* is the most probable word in Topic 2. This ranking is derived from the ϖ matrix of the topic model.

**Table 8:** **Selected genres**.

Topic 1	Topic 2	Topic 3	Topic 4	Topic 5	Topic 6
Film	Evil	Killed	Scientist	Frank	Town
Documentary	King	Gang	Professor	Max	Small
la	Princess	Revenge	Thomas	Johnny	Local
di	Prince	Kill	Test	Prison	Dead
Music	Village	Boss	Project	Plane	Body
Interviews	Helen	Death	Research	fbi	Accident
Films	Land	Gun	Truth	Stop	Sees
Series	Powers	Named	Experience	Terrorists	Car
Footage	Queen	Brother	Mind	Led	Road

Table 8 appears to be fairly coherent. For example, Topic 1 could be considered to be about the film or music industry. Topic 2 consists of word often associated with fantasies or fairy tales. Topic 3 is made up of words consistent with action/revenge films. Topic 4 lists science words, and so on for the other genres. It is plausible to consider that synopsis-based topics might be more effective at predicting human annotated genres than content-based features or likability-based topics.

## Study 5: Predicting with Synopsis-Based Topics

We conducted Study 5 to further investigate the alternative hypothesis that the poor predictive power of content-based features in Study 3 is attributable to a large feature space of 1,420 features. Given the seemingly coherent categories that emerged from a topic model of synopses in Study 4, we decided to create a predictive model using as input the synopsis-based topics (Study 4) and compare its performance to the content-based models (Study 3) and the likability-based topic model (Study 2). This three way comparison allows us to assess the relative impact of topic models vs. the relative impact of content-based features.

### Method

#### Data

We used the same 9,249 matched films and associated IMDB genre as was used in Study 2 and 3. The synopsis-based topics created in Study 4 was used as input for the predictive model.

#### Procedure

As in Studies 2 and 3, we used the WEKA toolkit (Hall et al., 2009) to build a set of predictive models. Congruent with Study 2, each model uses as features only the distribution of topics associated with each film, which is a row vector from the topic model in Study 4. As in Studies 2 and 3, the genre class to be predicted is the first genre listed by IMDB. In order to be directly comparable to Study 2, the same set of five models was used, i.e., ZeroR, NaiveBayes, AdaBoostM1, J48, and IBk, with the same set of parameters. Likewise each model was trained using 10-fold cross validation, and significant differences were measured using a corrected paired samples t-test, *p* = 0.05.

### Results and discussion

The results of the synopsis-based predictive models are displayed in Table 7 along with the parallel results from Studies 2 and 3. Numbers shown indicate percent correct, aggregated across all genre categories. All significant differences are relative to the ZeroR model for each set using a paired samples t-test, *p* < 0.05.

Performance on the synopsis-based models is comparable to the content-based models and worse than the performance on the likability-based models. Synopsis-based models have little deviation away from ZeroR. Moreover, the highest performing synopsis-based model, IBk, has roughly 30% accuracy, virtually identical to the content-based J48 model’s accuracy of 29%. All differences are significant, except AdaBoostM1, which is identical to ZeroR.

With regards to our hypothesis for this study, there does not appear to be a particular advantage in using a topic model to create synopsis-based predictive model. The predictive power is approximately the same as the content-based model, which used 1,420 features, nearly all of them created by tf*idf from the synopsis. In this study, reducing the dimensionality of the feature space for synopses does not improve prediction of human annotated genres, but it does not hurt either.

## Study 6: User-Viewed Topics

In this final study, we investigated the properties that make likability ratings so effective at forming coherent genre categories. Topic models essentially capture co-occurrence data of words across multiple documents, or in the present case, co-occurrence data of films across multiple users. In Study 1, co-occurrence data was weighted by frequency, i.e., the number of stars given to a film. The question we address in the present study is whether frequency information, which represents likability, is essential to topic formation. If topics still emerge when likability information has been stripped away, then likability cannot correspond to the theoretical notion of an ideal (Barsalou, 1985). We call the topics created without frequency information user-viewed topics, because they only capture what films have been seen by a user (a binary feature).

### Method

#### Data and procedure

The same Netflix dataset was used as in Study 1. However, the mapping procedure was altered to remove frequency information. As in Study 1, each customer is a mixture of genres, and each genre is a distribution over films. To transform the existing Netflix dataset using this mapping, we collected all of the films seen by a customer. However, rather than letting the number of stars given to that film represent the number of times that film’s label appears, we let the label appear only one time. For example, if a customer had only rated the film *Whale Rider* and had given it three stars, then the customer would be represented as (Whale Rider), analogous to a document containing the same word only once. Under the assumptions of this mapping and the underlying topic model, each star in a customer’s rating can be generated by a single genre. This mapping is in contrast to Study 1, where the customer would be represented as (Whale Rider, Whale Rider, Whale Rider) and each star could be generated by a different genre.

The inference parameters for the topic model in Studies 1 and 4 were also used in the present study. The number of topics was 50, the prior for topics appearing in a document (α) was 1, and the prior for words appearing in a topic (β) was 0.01. The model was run for 200 iterations.

### Results

The topics found by the model are displayed in Table 9. The topic lists are rank ordered by probabilistic membership derived from the ϖ matrix of the topic model, e.g., *Flowers of Shanghai* is the most probable film of Topic 1.

**Table 9:** **Selected topics**.

Topic 1	Topic 2	Topic 3	Topic 4
Flowers of Shanghai	Amour de Femme	The Omen	The Ballad of Little Jo
The Last Ride	A Matter of Dignity	The Deviants	Bionicle: Mask of Light
Food of Love	The Greatest American Hero	Dark Shadows Reunion	Star Trek
Santana: Sacred Fire	Bill Maher: Victory Begins at Home	Parineeta	Devdas
Parting Shots	Thumbelina	Shelter Island	Jacob’s ladder
Amelie: Bonus Material	Paradise lost 2: Revelations	Smiley’s People	Laadla
The Dead Zone: Season 3	Infinity	The Story of Us	Wing and a Prayer
The Einstein of Sex	Fugazi: Instrument	Frostbite	Alien Visitor
Billy Blanks: Tae Bo	James Brown Live: House of Blues	Terminator 2: Bonus Material	Wasabi
The Fluffer	WWE: No Way Out	Blade: Trinity	Social Distortion

It is difficult to, see any coherent structure in Table 9. For example, Topic 1 contains horror, exercise, music, and action films. Likewise Topic 2 contains drama, comedy, music, and wrestling titles. Topic 3 contains horror, comedy, drama, and action, and so on for Topic 4. Clearly the coherence of user-viewed topics has been severely impaired by removing frequency information. This result demonstrates that likability ratings are necessary for the emergence of genre; simply tracking whether or not a user has viewed a film is insufficient. This finding further supports the relationship between likability ratings and the notion of ideals in film genre.

## General Discussion

The work reported in this paper was concerned with only one kind of category, film genre, and two general approaches to predicting genre using either likability ratings or content-based features. There were three basic findings from the six studies presented. First, when likability ratings are input to a topic model, film genres emerge. The internal structure of these likability-based topics is coherent, and there is a one to one correspondence between likability-based topics and film genre for many films. This effect disappears when likability is collapsed into a binary value indicating whether a user has or has not viewed a film. Thus genres emerge as a result of likability (a post-viewing measure) rather than choice (a pre-viewing measure). As a result, our likability ratings have non-trivial similarity with rating items according to ideals (Barsalou, 1985). Since the ideal is likability, however, ideals are implicit: participants may use their own criteria for deciding how much they liked the film.

The second basic finding in this work is that likability-based topics can predict human annotated genres with 41% accuracy, approximately twice the base rate performance of 23% accuracy. The 41% performance is based on a single genre classification, when IMDB allows multiple classifications. So 41% performance represents the lowest, most conservative figure. Moreover, when the likability-based topics are transformed into a unary vector representing the single most probable topic per film, accuracy only decreases by 2%, indicating a one to one relationship between likability-based topics and film genres for many films. That a content-free analysis, based purely on likability ratings, can predict genres is surprising and provocative. Even more surprising is that likability-based genres can predict so well, especially given the stringent single-class criterion of correctness.

Our multiple investigations comparing likability-based models and content-based models led to the third basic finding of our research, which is that likability-based models have greater predictive power for human annotated genres than do any of the intuitive content-based features we tested. We established this result initially in Study 3, using a variety of content-based features including real-world information (director, actors, rating) and film-internal information (synopsis). In later studies we considered alternative explanations, including the possibility that the reduced dimensionality of the feature space was the reason for the likability-based genre model’s success. However, a three way comparison between likability, content, and synopsis based models allowed us to compare the differential impact of three factors, namely topics, ratings, and content as shown in Table 10.

**Table 10:** **Summary of hypotheses in studies 2–5**.

Model	Topics	Ratings	Content
Likability-based (LB)	+	+	−
Content-based (CB)	−	−	+
Synopsis-based (SB)	+	−	+

Each row in Table 10 refers to one of the three models, likability-based, content-based, and synopsis-based, created in Studies 2, 3, and 5 respectively. Each column of Table 10 corresponds to a salient dimension of the models, i.e., the use of a topic model, likability ratings, and content-based features when predicting genre. Recall that the predictive accuracy of likability-based genres was approximately 41% and the other two models significantly lower at approximately 29%.

The most interesting comparisons in Table 10 involve the dimensions of topics and ratings. In Table 10, topics do not appear to be contributing to predictive accuracy, given that *LB* > *SB*. However, ratings do appear to affect predictive accuracy, *LB* > (*CB* = *SB*). In other words, despite the coherent topics that emerged from synopses in Study 5, topics did nothing to improve predictive performance.

A limitation to these results is that only a small number of content-based features and models were compared. Thus, it could be the case that some untested content-based feature could yield different results. However, it is worthwhile to consider why content-based (including synopsis) models might be poor at predicting human annotated genres. Why doesn’t a topic like Topic 2 in Table 8, by containing words like (evil, king, princess, prince) successfully predict genres like fantasy? Perhaps for the same reason that other content-based features fail: the films *Shrek*, *The Princess Bride*, and *The Man in the Iron Mask* intuitively match this topic but are from three different genres. Likewise, while it might be plausible to use Sylvester Stallone as a feature for action films, there are also exceptions, like his role in the animated film *Antz*. In short, as is highly familiar to genre theorists, the setting of a film, who directed it, etc., are not as important to determining the genre of a film as is the overall effect of the film on the audience, e.g., a zombie film that induces laughter is a *comedy*. If film genre is a goal-directed category structured around ideals, like *birthday present*, then a content-based analysis will always fail because what matters is the effect on the viewer – something a content-based analysis cannot capture.

What would cause Netflix users to organize film genres around ideals? There is some evidence to suggest that ideals, which are centered on goals, are inextricably tied up in emotion. For example, 8 out of 18 of the ideal dimensions used by Barsalou (1985) involve liking, enjoyment, and emotion. In addition to the more general role that emotions may play in ideals, the centrality of emotion in the experience of film has been argued (Smith, 2003). Thus there is reason to believe that the emotional aspect of film might be more salient to viewers than specific features of the film. It also appears that we structure our perception of emotional communication, such as facial expression of emotion, around ideals rather than family-resemblance (Horstmann, 2002). So if our goals in watching film involve specific emotional experiences for ourselves and if films are crafted to evoke emotional responses, then Netflix users could be expected to use liking, enjoyment, and emotion as ideals to structure film categories. Our results support the conclusion that film genres are structured more around these implicit ideals than around content-based features of film.

Our approach to genre and film diverges from the common methodologies for investigating category structure (Murphy, 2004) because we wished to avoid assuming categories *a priori*. This precluded using any methodology that compared a category to its items. Because we avoided assumptions of categories, our findings have implications for existing genre studies in film. Recall from the introduction the problems of circularity and the monolithic assumption (Stam, 2000). The basic problem of circularity lies in a supervised approach in which a critic tries to align film features with a given genre category. A likability-based model, as an unsupervised model, avoids this problem entirely because there is no initial assumption of genre used to define the features of genre. Instead, genre emerges from genre-agnostic likability ratings. The second problem of definition, the monolithic assumption, is addressed by the structure of the topic model. Under this model, every film has some probability of membership in every genre. Study 2 above illustrates that it is not necessary to pigeonhole a film into a genre in order to create meaningful genres: even using a probabilistic definition of genre, one can still approximate the monolithic assumption to 41% accuracy. Pluralistic genres, like *romantic comedy*, are not a special case but are represented in the same way as any other genre.

Using the likability-based definition of genre, we can also clarify problems of film genre analysis that have been raised by theorists (Stam, 2000). First, are genres real or imagined? According to our approach, genres are only manifested through people’s preferences and not by the content-based features of the work. Therefore they do not have any status in the world except as a consensus of preferences across large groups of people. Secondly, theorists have asked if the number of genre categories is finite or infinite. The structure of the topic model suggests that the number of genres is completely arbitrary, and is controllable using the parameter *T*, the number of topics. Our model therefore allows for an arbitrarily coarser or finer hierarchy. As a result the number of genres is limited to the specificity of viewer preferences. If viewers become more or less discriminating in their ideals, the structure of genres will change. Thirdly, on whether genres are timeless or are trendy, the likability-based model suggests that they are trendy. Any new ratings that are assimilated into the model can change the resulting genres. This property allows for genres to change over time, to be adapted, and extended in new ways, e.g., *space western*, and to disappear. Finally, as to whether the genres are universal or culture bound, one can speculate that they are culture bound to the extent that one culture may rate films according to a different set of ideals. Thus our analyses lend empirical support to post-structuralist views of film genre that reject the role of defining features, or necessary and sufficient conditions, that plague earlier structuralist accounts:

My argument about text classes or genres can be summarized as follows: Classifications are empirical, not logical. They are historical assumptions constructed by authors, audiences, and critics in order to serve communicative and esthetic purposes. Such groupings are always in terms of distinctions and interrelations, and they form a system or community of genres. The purposes they serve are social and esthetic. Groupings arise at particular historical moments, and as they include more and more members, they are subject to repeated redefinitions or abandonment (Cohen, 1986, p. 210).

Models of categorization in cognitive psychology (Collins and Quillian, 1969; Schaeffer and Wallace, 1970; Smith et al., 1974; Rosch, 1975, 1978) have largely been driven by behavioral data where participants are asked whether an item is related to a category. The corresponding responses are often ratings made by the participant or their reaction time in a decision task. Likability ratings present an alternative methodology: allow the participants to make ratings without priming them with a category and without reference to a particular set of features. While the prototype theory of categorization (Rosch, 1975, 1978) does not necessarily make assumptions about the internal structure of the items, when operationalized into a classifier, notions of family-resemblance seem to require a feature-based internal structure (Smith and Medin, 1981). Our model, in contrast, requires only storing the name of the film and whether the viewer liked it. No film-internal structure, no features, are considered or stored by the model. The likability-based topics produced by the topic model do not need to be stored and can instead be viewed as the product of a continuous process. The IBk classifier, which classifies a new instance based on the majority genre of its nearest neighbors, can be viewed as a mapping from an individual’s ideals of likability to the linguistic label commonly used in their community. Although likability ratings appear to avoid problems of internal structure when applied this way, one could argue that they do not fully explain the phenomenon because the question of *why* individuals produce different ratings has not been directly addressed. In contrast our model only assumes that people like some genres more than others and express this in their ratings of film.

In summary, likability-based topics offer a novel and useful way of considering film genre. Rather than being a taxonomic set of categories determined by family-resemblance, film genre appears to be based on our ideals of enjoyment. These ideals, which vary from person to person, are consistent enough across hundreds of thousands of people for traditional genres to emerge from likability ratings. One possible explanation for this consistency is that likability is based on some universal constants of emotion. However, likability-based genres as described in this paper do not represent a complete theory of film categorization. In order to understand this phenomenon fully, it is necessary to understand how the ratings themselves are generated.

## Conflict of Interest Statement

The author declares that the research was conducted in the absence of any commercial or financial relationships that could be construed as a potential conflict of interest.

## Footnotes

^1^http://andrewmolney.name
